# A Survey of Periodic Dental Visits Among Patients Receiving Preoperative Consultations

**DOI:** 10.14789/ejmj.JMJ24-0040-CR

**Published:** 2025-04-03

**Authors:** YOSHIKO YAMAMURA, RYO UMEYAMA, JUNKO YAMAZAKI, SHIHO KOROKU, SHUNSUKE NAMAKI, SEIJI ISHIKAWA, MITSUYO SHINOHARA

**Affiliations:** 1 Oral and Maxillofacial Surgery, Juntendo University Hospital, Tokyo, Japan; 1 Oral and Maxillofacial Surgery, Juntendo University Hospital, Tokyo, Japan; 2 Department of Oral and Maxillofacial Surgery, Faculty of Medicine, Juntendo University, Tokyo, Japan; 2 Department of Oral and Maxillofacial Surgery, Faculty of Medicine, Juntendo University, Tokyo, Japan; 3 Department of Oral and Maxillofacial Surgery, School of Dentistry, Nihon University, Tokyo, Japan; 3 Department of Oral and Maxillofacial Surgery, School of Dentistry, Nihon University, Tokyo, Japan; 4 Department of Anesthesiology and Pain Medicine, Faculty of Medicine, Juntendo University, Tokyo, Japan; 4 Department of Anesthesiology and Pain Medicine, Faculty of Medicine, Juntendo University, Tokyo, Japan

**Keywords:** perioperative oral management, regular dental visits, preoperative outpatients, oral conditions

## Abstract

**Objectives:**

The aim of this study was to examine the correlation between regular dental visits and the oral health status.

**Design:**

This was a retrospective study.

**Methods:**

We included 3,138 patients who visited the preoperative outpatient clinic and underwent oral examinations between April and September 2020. Patients whose last dental visit occurred less than one year prior to data collection constituted the regular visit group, while the irregular-visit group comprised those with a last dental visit beyond one year prior to data collection. We examined the following information: last dental visit, sex, age, disease causing hospitalization, frequency of daily brushing, presence or absence of moving teeth, and oral hygiene status.

**Results:**

The frequency of brushing was lower in the irregular-visit group than in the regular-visit group, suggesting a lower awareness of oral health in the irregular-visit group. Furthermore, the oral hygiene status in the regular-visit group was better than that in the irregular-visit group, with more patients showing no tartar deposition.

**Conclusions:**

Regular dental checkups can mitigate perioperative complications, preventing them from adversely affecting the treatment of the underlying disease. It is crucial to emphasize the importance of oral management, explain the need for it, and actively encourage regular dental visits.

## Introduction

In April 2012, a revision of medical fees established “perioperative oral function management” to reduce complications after surgical procedures, such as postoperative aspiration pneumonia, and to improve the treatment of oral mucositis and oral infections associated with cancer chemotherapy and radiation therapy^1)^. In our hospital, a preoperative outpatient clinic was established in May 2019 for safe anesthesia management and to reduce postoperative complications in patients scheduled to undergo surgery or examination under anesthesia managed by anesthesiologists. In addition to anesthesiology consultation, checking drug being taken and admission explanation, dental hygienists screened the oral cavity to evaluate the presence or absence of upset teeth and the state of oral hygiene as a part of this initiative. Among the patients who were screened, some had moving teeth or extremely poor oral hygiene and had not visited a dentist for a long time. Limited studies have examined the correlation between regular dental visits and oral health status. Thus, we conducted a survey among preoperative outpatients to assess their adherence to regular dental visits.

## Materials and Methods

This study included 3,138 patients who visited our hospital’s preoperative outpatient clinic and underwent oral screening during a 6-month period from April to September 2020. The regular-visit group included patients whose last dental visit was less than one year prior to data collection, while the irregular visit group included those whose last dental visit was more than one year prior to data collection^2)^. We compared sex, age, disease causing hospitalization, daily brushing frequency, presence or absence of upset teeth, and oral hygiene status between groups. The presence of upset teeth was defined as the presence of one-to-three-degree motion teeth (Miller’s classification). Oral hygiene was assessed on three levels: “no problem” if no plaque or tartar was detected, “plaque” if visible plaque was observed, and “tartar” if visible tartar was observed. In addition, we examined the history of checkups in patients who visited the dental clinic before surgery and compared it with the national averages. Statistical analysis was performed using the chi-squared test or Student’s t-test, with significance set at p < 0.05.

This study was reviewed and approved by the Clinical Research Ethics Review Committee of our hospital (Approval No. E23-0434). Written informed consent was obtained.

## Results

### Periodic medical examination rates

**Figure 1:**
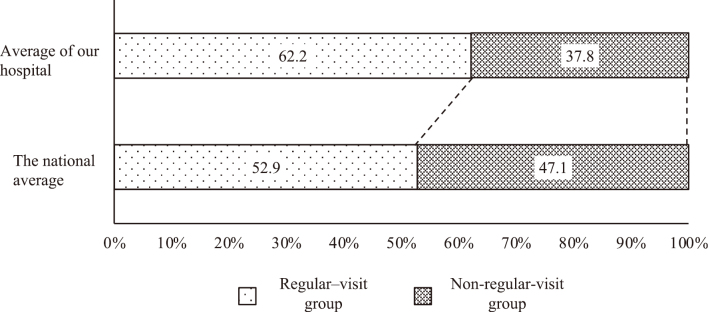
Periodic dental examination rate comparison with the national average The percentage of patients who underwent regular checkups within one year was higher than the national average among those who visited our preoperative outpatient clinic.

Among the patients, 1,953 patients (62.2%) received regular checkups in less than one year, while 1,185 patients (37.8%) did not receive any checkups (Figure 1). The percentage of patients who underwent regular checkups within one year was higher than the national average among those who visited our preoperative outpatient clinic^2)^.

### Patient background

Among the 3,138 patients included in this study, 1,490 were males and 1,648 were females, ranging from 0 to 95 years old, with a mean of 54.7 ± 21.1 years. The mean age was higher in the regular- visit (55.9 ± 21.4 years) than in the irregular-visit (52.9 ± 20.4 years) group (p < 0.05: student’s *t*-test). In the regular checkup group, there were 940 males and 1,013 females, while the irregular checkup group comprised 550 males and 635 females. The number of patients with upset teeth was 554 (28.4%) in the regular-visit group and 323 (27.3%) in the irregular-visit group. No significant differences were observed in terms of sex or the presence or absence of upset teeth between groups (Table 1). The diseases causing hospitalization in the regular-visit were lung cancer, breast cancer, and uterine fibroids in that order, while the diseases in the irregular-visit were breast cancer, lung cancer, and uterine fibroids in that order.

**Table 1:** Patient background

	Regular–visit group	Irregular-visit group	
Mean age (years)	55.9 ± 21.4 (range : 0-93)	52.9 ± 20.4 (range : 2-95)	p < 0.05 (student’s t-test)
Gender (male : female)	940 : 1013	550 : 635	N.S. (chi-squared test)
Upset tooth (presence : absence)	554 : 1399	323 : 862	N.S. (chi-squared test)

### The number of times a day to brush

**Figure 2:**
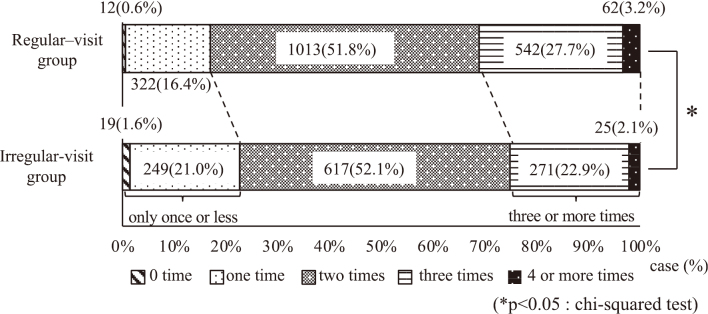
The number of times a day to brush In the regular-visit group, 604 (30.9%) patients brushed three or more times, accounting for approximately one-third of the total number of patients. In the irregular-visit group, 296 (25.0%) patients brushed three or more times, accounting for approximately one-fourth of the total, and 268 (22.6%) brushed only once or less, accounting for approximately one-fourth of the total. There was a significant difference in the proportion of patients in each group (p < 0.05, chi-squared test).

In the regular-visit group, 542 patients (27.7%) brushed three times, while 62 (3.2%) brushed four or more times, accounting for 604 patients (30.9%) who brushed three or more times (approximately one-third of the total patient population). Conversely, within the irregular-visit group, 271 patients (22.9%) brushed three times, and 25 patients (2.1%) brushed four or more times, accounting for 296 (25.0%) patients who brushed three or more times (approximately one-fourth of the total). Nineteen patients (1.6%) brushed zero times, 249 (21.0%) brushed once, and 268 (22.6%) brushed less than once, accounting for about one-fourth of the total number of patients. There was a significant difference in the proportion of patients in each group (p < 0.05, chi-squared test) (Figure 2).

### Oral hygiene

**Figure 3:**
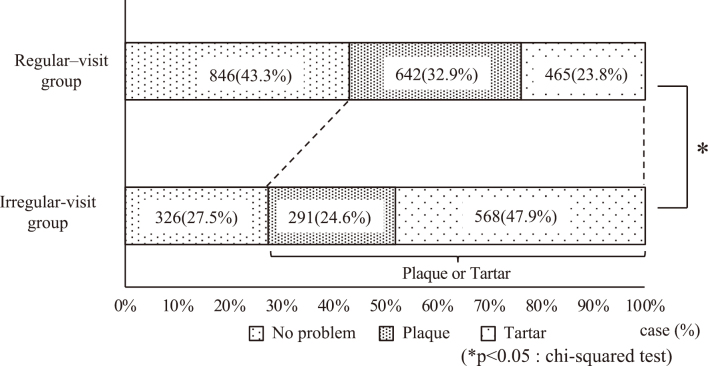
Oral hygiene More patients in the regular-visit group had “no problems” than those in the irregular-visit group. Approximately half of the patients in the regular-visit group had tartar deposits. There was a significant difference in the proportion of patients in each group (p < 0.05, chi-squared test).

In the regular-visit group, 846 patients (43.3%) had “no problem,” 642 (32.9%) had “plaque,” and 465 (23.8%) had “tartar,” indicating that “no problem” was more prevalent in this group than in the irregular-visit group. In the irregular-visit group, 326 patients (27.5%) had “no problem,” 291 (24.6%) had “plaque,” and 568 (47.9%) had “tartar,” indicating that approximately half of the patients in the irregular-visit group had tartar deposition. The number of patients with dental plaques was higher in the regular group than in the irregular group. There was a significant difference in the proportion of patients in each group (p < 0.05, chi-squared test) (Figure 3).

## Discussion

In April 2012, our country’s reimbursement system incorporated “perioperative oral function management” to reduce postoperative complications and prevent the development of oral mucositis and infections associated with cancer chemotherapy and radiation therapy^1)^. Initially, patients were referred by their respective departments to ours for oral screening before initiation of treatment for the underlying disease; however, from May 2019, a dedicated “preoperative outpatient clinic” was established for surgery or examination under anesthesia. In the preoperative outpatient clinic, oral screening is conducted by a dental hygienist, in addition to an anesthesiology consultation, checking drug being taken and an admission explanation. This evolution in the approach enhances the comprehensive care provided to patients. The presence of upset teeth, oral hygiene, and dental problems were evaluated, and if preoperative dental intervention was deemed necessary, the patient was examined and treated by a dentist. Within the preoperative outpatient clinic, a specific number of patients were identified with preoperative dental issues, including moving teeth and poor oral hygiene. Given the assumption that these patients likely did not undergo regular dental examinations, this study was conducted to investigate the actual situation with regards to routine dental checkups.

As per a 2020 survey conducted by the Japan Dental Association, 33.8% of the population received regular dental checkups^3-5)^. This marks an increase compared to the data from the 2018 survey. Additionally, data from the 2016 National Health and Nutrition Survey revealed that the “percentage of persons aged 20 years or older who received a dental checkup in the past year” was 52.9%, indicating a notable increase over time^2)^. The rate was higher (62.2%) among our preoperative outpatients. This difference may be due to the high level of interest in dental diseases among our patients or the fact that our physicians routinely educate patients about the importance of dental examinations.

There were no significant differences in sex or the presence or absence of moving teeth between the regular- and irregular-visit groups. However, the average age of the regular-visit group was higher than that of the irregular-visit group, and they brushed more frequently. This was presumably due to the progression of periodontal disease and dental caries with age, which increases the awareness of dental diseases and oral health. In the irregular-visit group, tartar deposits were found in 568 (47.9%) patients and required removal before surgery. The regular-visit group had more plaque than the irregular-visit group, but there was no significant difference in the number of upset teeth, suggesting that even the regular-visit group was expected to have inadequate brushing after the visit, and that the dentist indicated the need for regular oral management. There had also been reports that patients over the age of 75 often had oral problems such as upset teeth and poor oral hygiene^6)^. So it thought that they were aware of the need for oral management. The degree of care required by the patient and the family structure was unknown in this study. In addition, the most frequent diseases that required hospitalization were malignant tumors such as lung cancer and breast cancer. There is a possibility that additional treatment will be required after surgery or examination, so the importance of regular oral management increases even further.

It has been reported that 28.7% of Japanese individuals who interrupted regular dental visits because of the coronavirus disease outbreak had an exacerbation of periodontitis^7)^. In addition, patients undergoing thoracic surgery who receive regular dental examinations have a lower risk of developing complications, such as postoperative pneumonia, than those who do not receive dental examinations^8)^. Patients with multiple advanced caries are also at a higher risk of developing postoperative pneumonia^8)^. Irregular dental visits and multiple caries increase the risk of postoperative pneumonia. Thus, although the importance of regular dental checkups is clear, many citizens do not receive such checkups. It is necessary to educate patients with systemic diseases to regularly visit dentists and maintain a healthy oral environment. Additionally, if the time between the preoperative outpatient visit and surgery is relatively short, minimal dental treatment should be administered to avoid dental problems during the perioperative period. Considering this situation, we believe that the importance of periodic dental visits should be recognized by the public.

In June 2022, the government approved the “Basic Policies for Economic and Fiscal Management and Reforms 2022” by a Cabinet decision, which included the promotion of a lifelong dental health checkup system for all generations of citizens. In the future, annual dental checkups may become mandatory for all citizens. As the average life expectancy has increased in recent years, patients with systemic diseases are expected to have more opportunities to receive treatment for their underlying diseases. Therefore, we believe that regular dental checkups should be recommended because they can prevent dental problems before the treatment of the primary disease and facilitate treatment.

A notable limitation of this study was the inability to track whether postoperative patients received education regarding the importance of regular checkups. A recommendation for future research includes a longitudinal follow-up report to address this aspect comprehensively.

In conclusion, the proportion of patients who visited our preoperative outpatient clinic and had a dental visit within less than one year was 62.2%, indicating a greater interest in dentistry than the national average. The age and number of times brushed per day were higher in the regular-visit group than in the irregular-visit group. No significant differences were observed in terms of sex or presence or absence of moving teeth. Regarding the oral hygiene status of the regular-visit group, fewer patients had tartar deposits compared to those in the irregular-visit group. In essence, regular dental visits can prevent preoperative dental problems and facilitate treatment. Therefore, it is imperative to explain the importance of oral management and educate patients about the importance of regular dental visits.

## Funding

No funding was received.

## Author contributions

YY designed and executed the experiments and wrote the manuscript. RU, JY, SK, SN and SI contributed to the concept and helped to write the manuscript. MS is a co-supervisor and edited the manuscript. All authors reviewed and approved the final manuscript.

## Conflicts of interest statement

The authors declare that there are no conflicts of interest.
